# Effects of TiB_2_ Particles on the Microstructure Evolution and Mechanical Properties of B_4_C/TiB_2_ Ceramic Composite

**DOI:** 10.3390/ma14185227

**Published:** 2021-09-11

**Authors:** Haiyan Niu, Yu Zhu, Ning You, Yangwei Wang, Huanwu Cheng, Dujun Luo, Mengying Tang, Jiamin Zhang

**Affiliations:** 1 Science and Technology on Complex and System Simulation Laboratory, Beijing 100072, China; niuzjs@126.com (H.N.); ningyou2021@126.com (N.Y.); 2 Beijing Institute of Aerospace Control Devices, Beijing 100039, China; 1055480577@163.com; 3 School of Materials Science and Engineering, Beijing Institute of Technology, Beijing 100081, China; chenghuanwu@bit.edu.cn (H.C.); dujun2184@163.com (D.L.); tangmengying1996@163.com (M.T.); zhangjiamin1996@163.com (J.Z.); 4 National Key Laboratory of Science and Technology on Materials under Shock and Impact, Beijing 100081, China

**Keywords:** pressureless sintering, B_4_C-TiB_2_, particle size, microstructure, microstructure evolution

## Abstract

B_4_C/TiB_2_ ceramic composites reinforced with three size scales (average particle size: 7 μm, 500 nm, and 50 nm) of TiB_2_ were prepared by using a pressureless sintering furnace at 2100 °C under Ar atmosphere for 60 min. The results demonstrated that during the sintering process, TiB_2_ located on the boundaries between different B_4_C grains could inhibit the grain growth which improved the mass transport mechanism and sintering driving force. A semi-coherent interface between B_4_C and SiC was found, which is supposed to help to reduce the interface energy and obtain good mechanical properties of the B_4_C/TiB_2_ ceramic composite. On sample cooling from sintering temperature to room temperature, the residual tensile stress fields formed at the TiB_2_ interfaces owning to the thermo-elastico properties mismatched, which might have contributed to increase the ability of the sample to resist crack propagation. The results showed that the relative density, Vickers hardness, and fracture toughness of the composite with 20 wt.% submicron and 10 wt.% nano-TiB_2_ were significantly improved, which were 98.6%, 30.2 GPa, and 5.47 MPa·m^1/2^, respectively.

## 1. Introduction

Boron carbide (B_4_C) ceramics are interesting structural ceramics in view of their outstanding physical and mechanical properties, especially the combination of low density and extremely high hardness which make them superior anti-ballistic materials over other armor ceramics (such as Al_2_O_3_, SiC) [1,2,3]. However, the expensive costs of B_4_C ceramics fabricated through the hot isostatic pressing (HIP) method severely limits its wide application in the armor protection field [4,5,6]. In addition, its low self-diffusivity efficiency indicates that the sintered body could not achieve the goal of densification through the single solid-state sintering technique. Recently, numerous attempts have been made to overcome these disadvantages, such as the introduction of a second phase and sintering additives into the B_4_C matrix to fabricate composites. Transition metal borides, such as TiB_2_ [7,8], ZrB_2_ [9,10], and HfB_2_ [11], having high thermal expansion coefficients, and the residual stress fields between B_4_C and borides rising during the cooling process possibly enhance the fracture toughness of the fabricated composites [12,13].

Recently, the B_4_C/TiB_2_ ceramic composites have been the object of numerous works [14,15,16,17]. The additives of TiB_2_ to B_4_C phase can maintain the advantages of high Vickers hardness and low density of B_4_C and, in addition, inhibit the grain growth [16]. Additionally, the physical and mechanical properties of the B_4_C/TiB_2_ composites without additives prepared from the B_4_C and TiB_2_ powder are very low. The relative density of B_4_C-30 wt.% TiB_2_ composites without any additives prepared via pressureless sintering was lower than 90% [18,19]. The flexural strength of the B_4_C/TiB_2_ ceramic composite reached 717 MPa for the hot-pressured method, which was above two times higher than those (260–361 MPa) fabricated via pressureless sintering [16]. Many researchers have used the B_4_C-TiO_2_-C powder mixture to prepare the B_4_C/TiB_2_ composites in different ways, such as the reactive pressureless sintering, hot-pressing, and pulse electric-current sintering [20,21]. Since the introduction of fine TiB_2_ grains by in-situ reaction, the B_4_C and TiB_2_ grain size retained submicron sizes, and the mechanical property tests indicated that the prepared B_4_C/TiB_2_ ceramic composites achieved the excellent Vickers hardness ~39.3 GPa and flexural strength ~865 MPa, respectively [7]. B_4_C with various particle sizes was introduced to fabricate B_4_C/TiB_2_ ceramic composites under the condition of hot pressing, and both of the B_4_C and TiB_2_ grains were grown compared to the raw powders after hot pressing [22]. Many studies have shown that for B_4_C ceramic composites, C and Si are good sintering aids [23,24,25,26,27]. Carbon removes oxides (such as B_2_O_3_) in the B_4_C raw powder, and improves the interfacial tension by the way of solid solution of carbon atoms into the boron carbide lattice, which increases the sintering driving force [23,24]. A small amount of Si in the B_4_C ceramics tends to form a silicide phase, which could improve the sintering ability of B_4_C [25,26,27].

Although hot-pressing and pulse electric current sintering can obtain high-performance composites, the equipment and production costs are high, and the product size is small. The pressureless sintering is an efficient way to fabricate B_4_C/TiB_2_ composites with large sizes and low costs. At present, the research on improving the performance of the B_4_C/TiB_2_ ceramic composites prepared under pressureless sintering conditions is relatively scattered [18,28,29], and these reports indicate that the sintering temperature and TiB_2_ content have a great influence on the microstructure and density of the composite. Additionally, the research regarding the TiB_2_ particle size affecting the sintering behavior of B_4_C/TiB_2_ ceramic composites under pressureless sintering conditions is rarely reported. In our present work, the B_4_C/TiB_2_ ceramic composites with 30 wt.% TiB_2_ were fabricated via the pressureless sintering method from commercial B_4_C raw powder with the average size of 3 μm and TiB_2_ raw powder with three different size scales (7 μm, 500 nm, and 50 nm), and mixed in variable mass ratio. In addition, carbon black and silicon particles were used as sintering auxiliary components. Furthermore, the effect of TiB_2_ grains on the interfaces to optimize the microstructure of the B_4_C/TiB_2_ composites was thoroughly investigated. This research should be beneficial to fabricate the excellent performance of B_4_C/TiB_2_ ceramic composite.

## 2. Materials and Methods

**Figure 1:**
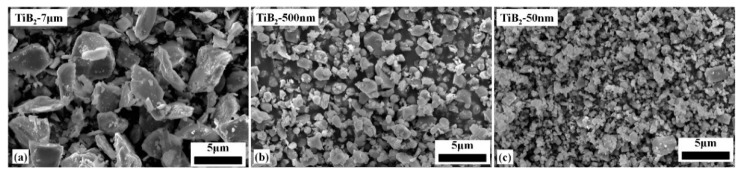
Nominal particle size and SEM images of commercial TiB_2_ raw powders (**a**) TiB_2_-7 μm (Micron), (**b**) TiB_2_-500 nm (Submicron), (**c**) TiB_2_-50 nm (Nano).

Raw materials were B_4_C powder (3 μm, purity: >99.5%; Zhengzhou Songshan Boron Technology Co., Ltd., Zhengzhou, China), silicon raw powder and carbon black raw powder (submicron, purity: >99.8%, Shanghai ST-NANO Co. Ltd., Shanghai, China), and TiB_2_ powder (purity: >99%; Shanghai ST-NANO Co. Ltd., Shanghai, China). Figure 1a–c show the three type morphologies of the TiB_2_ raw powders. Figure 1a depicts the microtopography of micro-TiB_2_ powder with average size about 7 μm. Figure 1b,c show the microtopography of submicron TiB_2_ powder with average size about 500 nm and nano-TiB_2_ powder with average size about 50 nm, respectively. Table 1 lists the phase composition of the three mixtures. The mixed raw powders were ball-milled in ethyl alcohol absolute with ZrO_2_ balls and then dried using a rotary evaporator (R205B, Shanghai Shensheng Technology Co. Ltd., Shanghai, China). The powder mixture was pressed in a graphite die and then cold isostatic pressed (CIP, LDJ100/320–300, Sichuan Aviation Industry Chuanxi Machine Factory, Sichuan, China) to form a green body with a 50 mm diameter. The samples were processed by pressurelss sintering in a graphite crucible (FCT Systeme GmbH, Rauenstein, Germany) at 2100 °C for 60 min at a heating rate of 10 °C per minute under flowing Ar atmosphere.

The relative densities of the samples were determined through Archimedes’ principle in deionized water. The average grain size was estimated by intercept method and more than 200 grains on the surface after polishing and thermally etching were measured. The flexural strength of the prepared specimens which were cut into the bars of 3 × 4 × 35 mm^3^ was tested on an electromechanical universal testing machine (INSTRON-5566, Norwood, MA, USA) of which the crosshead speed was 0.5 mm per minute and the span was 30 mm. The fractural toughness of the composites tested on bars (the size of 3 × 6 × 35 mm^3^), and a notch depth of 3 mm, was measured by the single-edge notched beam (SENB) test of which the crosshead speed was 0.05 mm per minute and the span was 24 mm. Vickers hardness measurement applied a load of 1 kg for 15 s to the sample surfaces on a hardness testing device (AHVD-1000, Shanghai Jujing Precision Instrument Manufacturing Co., Ltd., Shanghai, China). The phases and components were characterized by X-ray diffraction (D8 Advance, Germany). The microstructure was analyzed by a scanning electron microscope (SEM, Hitachi-S3400N, Hitachi, Tokyo, Japan) and a transmission electron microscope (TEM, Oxford INCAX-ACT, Oxford Instruments, Oxford, UK). The TEM sample of a selected specimen was prepared through conventional mechanical thinning and finished with precision ion polishing system machine (PIPS, Gatan-691, Pleasanton, CA, USA).

## 3. Results and Discussion

**Figure 2:**
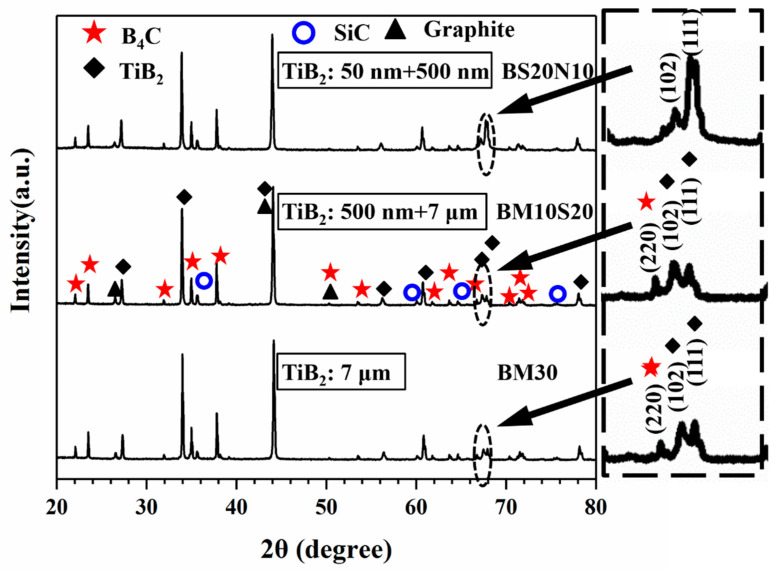
XRD patterns of B_4_C/TiB_2_ ceramic composites obtained with different grades of TiB_2_ powders.

The phase compositions of the sintered B_4_C/TiB_2_ ceramic composites with various raw powders are shown in Figure 2. All samples contained B_4_C, TiB_2_, SiC, and graphite. The X-ray characteristic peak patterns of the BM30 and BM10S20 were the same. With the introduction of TiB_2_ nanoparticle powders, the characteristic peaks of TiB_2_ changed significantly. For the BS20N20, the 2θ = 68.206° characteristic peak of the TiB_2_ was higher than the characteristic peak intensity of the sample BM30 and BM10S20. The well-defined peaks in the as-prepared B_4_C/TiB_2_ composite suggests that the TiB_2_ phase has a preferred orientation in (102) and (111).

**Figure 3:**
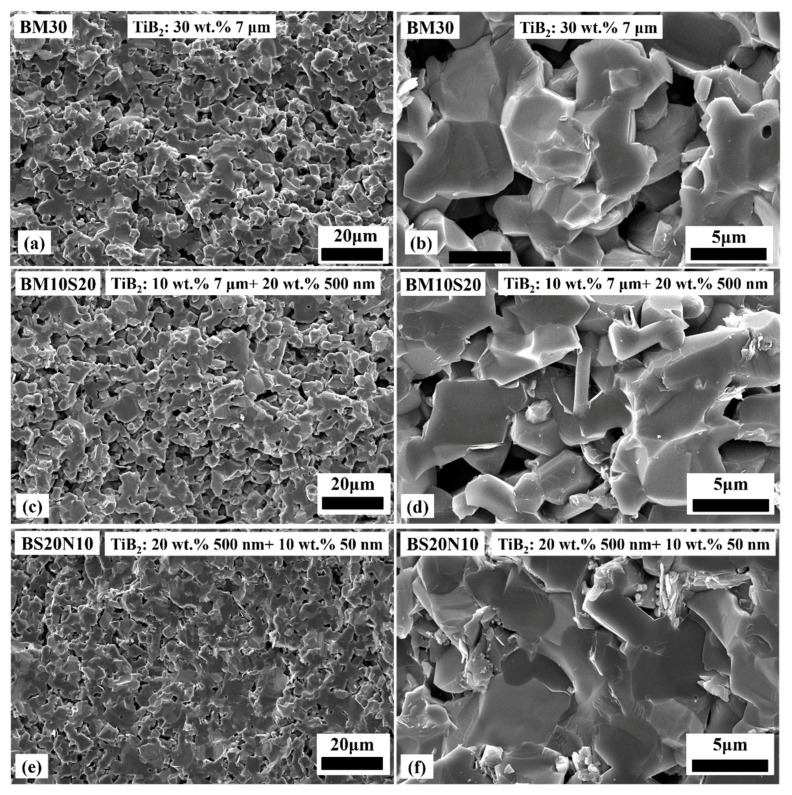
Fracture morphology SEM images of the B_4_C/TiB_2_ ceramic composites with various TiB_2_ raw powder mixtures. (**a**,**b**) BM30, (**c**,**d**) BM10S20 and (**e**,**f**) BS20N10.

**Figure 4:**
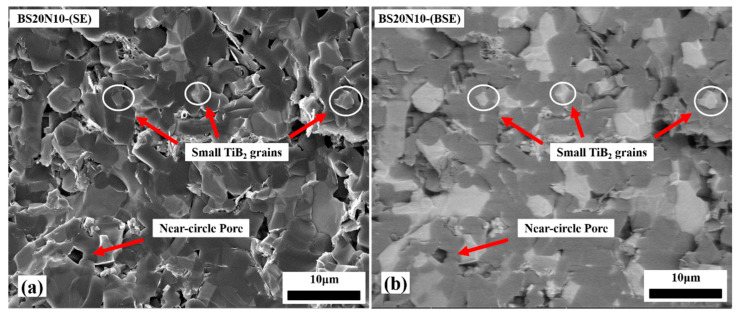
Small TiB_2_ grains in the B_4_C/TiB_2_ ceramic composite processed with a mixture of sub-micron and nano-sized TiB_2_ particles. (**a**) SEM image and (**b**) corresponding BSE image.

Figure 3 and Figure 4 show the SEM pictures of the fractured surface of the microstructure of the B_4_C/TiB_2_ ceramic composites. It could be clearly seen that due to the fact that the BM30 raw material powder particles are coarse and the sintering driving forcing is small, as shown in Figure 3a,b, there were a large number of interconnected open pores, and the coarsened particles were connected in an island chain. A large amount of sinter-necks with clear contours among the grains in the BM30 sample were still visible. With the size of TiB_2_ powder decreasing, the pore content and pore size decreased, and the dense areas increased significantly, as shown in Figure 3c,f. In the BS20N10 sample containing both 500 nm and 50 nm particle sizes of TiB_2_ powder, the shapes of the pores were relatively regular, tending to form regular polygon or nearly circular shapes, as shown in Figure 4a,b. Additionally, it can be inferred that these small particles belong to TiB_2_.

**Figure 5:**
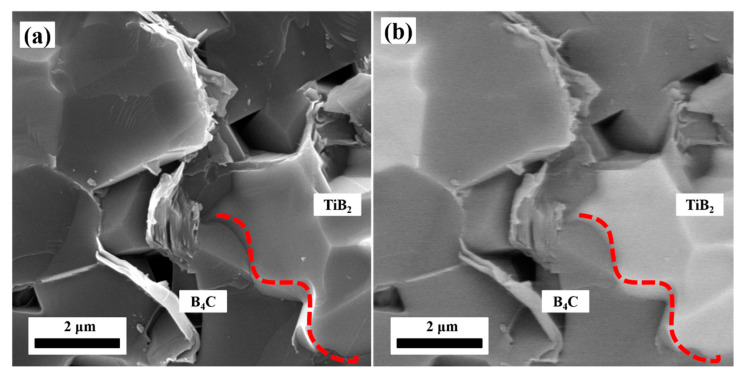
Interface between B_4_C and TiB_2_. (**a**) SEM image and (**b**) corresponding BSE image.

The TiB_2_ grains on the grain boundaries can pin the movement of the B_4_C grain boundaries and hinder the grain growth, thus increasing the content of grain boundaries and increasing the sintering rate [29]. In the BS20N10 sample, the interfaces between TiB_2_ and B_4_C phases are well distributed, indicating that TiB_2_ and other phases achieved good wetting during the sintering process as shown in Figure 5. The interface between B_4_C and TiB_2_ is jagged, which indicates that the interface feature helps to improve the ability to resist external loads.

**Figure 6:**
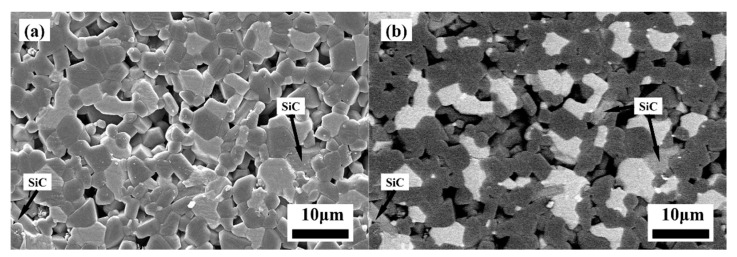
SEM images of the polished surfaces (**a**) and corresponding BSE images (**b**) in the sample.

**Figure 7:**
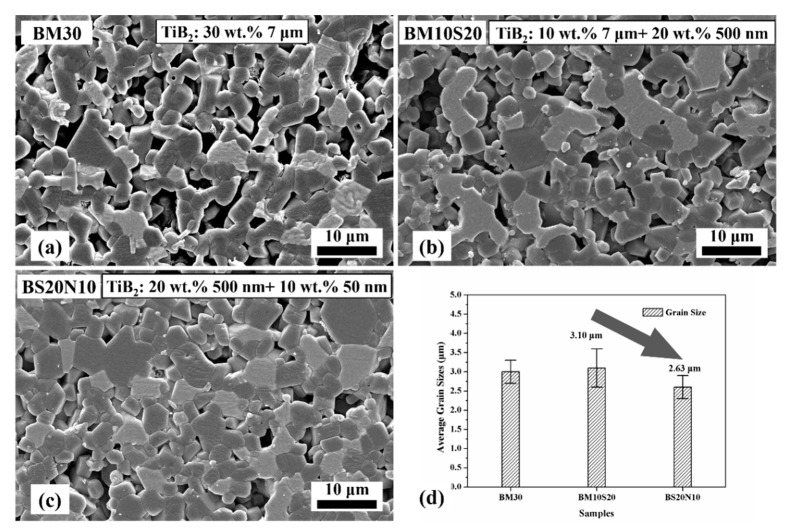
(**a**–**c**) SEM images of the polished surfaces and (**d**) plot of the average grain sizes in the samples.

The SEM pictures of the polished surfaces of the specimens sintered with various TiB_2_ powders are shown in Figure 6 and Figure 7. In Figure 6, SiC grains were dispersed and distributed on the B_4_C substrate in sample BS20N10, which acts as a pinning to prevent the grain boundary and inhibit grain growth. Additionally, the compound reaction of Si and C generated SiC exotherm, which helps the sintering process. Figure 7 show that the average grain sizes of the TiB_2_ in the prepared specimens with various raw TiB_2_ particles obtained under pressureless sintering conditions at 2100 °C for 60 min dwell were similar. B_4_C grains ranged from 2 μm to 10 μm, and comparing with the TiB_2_ raw powder with an average grain size of 50 nm, the grain sizes of the ceramic composites increased by a maximum of 200 times. With the size of TiB_2_ raw powder decreasing, the amount and size of the pores in the samples decreased significantly. The B_4_C average grain size of the BM30 sample to which the 7 μm-sized TiB_2_ powder was added was 3.01 μm, but many large pores were present in Figure 7a. B_4_C average grain size of the BM10S20 sample, with the TiB_2_ addition of 7 μm and 500 nm, was consistent with BM30, but the amount of the pores decreased, and the densification area of the BM10S20 sample increased. B_4_C average grain size of the BS20N10 sample with the TiB_2_ addition of the 500 nm and 50 nm remained close to the starting raw powder, about 2.63 μm, and the amount and size of the pores in the BS20N10 sample was significantly reduced. The relative density of the BS20N10 sample was also increased to 98.6%, as shown in Table 2.

**Figure 8:**
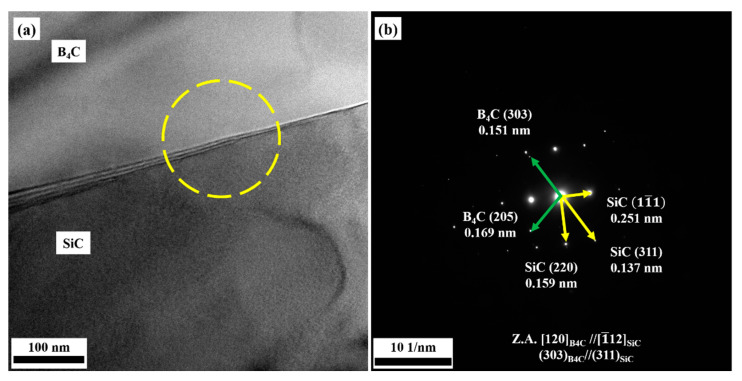
Interface between B_4_C and SiC. (**a**) Bright field TEM image, (**b**) SAED pattern.

Figure 8a–b are TEM images of the interface structure between B_4_C and SiC in the BS20N10 sample. According to the selected area-electron diffraction (SAD) result in Figure 8b, the unit cell structure parameter of B_4_C was a = b = 0.56 nm, c = 1.21 nm, α = β = 90°, γ = 120°, and the unit cell structure parameter of SiC was a = b = c = 0.44 nm, α = β = γ = 90°. The zone axis of the two phases of B_4_C and SiC satisfies the relationship: ${\left[120\right]}_{B_{4}C}//{\left[\bar{1}12\right]}_{SiC}$, and a group of crystal planes satisfies the relationship: ${\left(303\right)}_{B_{4}C}//{\left(311\right)}_{SiC}$. Additionally the interface between crystal plane ${\left(303\right)}_{B_{4}C}$ and crystal plane ${\left(311\right)}_{SiC}$ satisfies:

(1) $$\delta =\frac{d_{\left(303\right)}-d_{\left(311\right)}}{d_{\left(303\right)}}=\frac{0.151\mathrm{nm}-0.137\mathrm{nm}}{0.151\mathrm{nm}}=9.27\%$$

According to the calculation Formula (1), the mismatch degree between the crystal plane ${\left(303\right)}_{B_{4}C}$ and crystal plane ${\left(311\right)}_{SiC}$ is 9.27%, which could form a semi-coherent interface, and it helps to reduce the interface energy and obtains a bonding strong interface [30].

**Figure 9:**
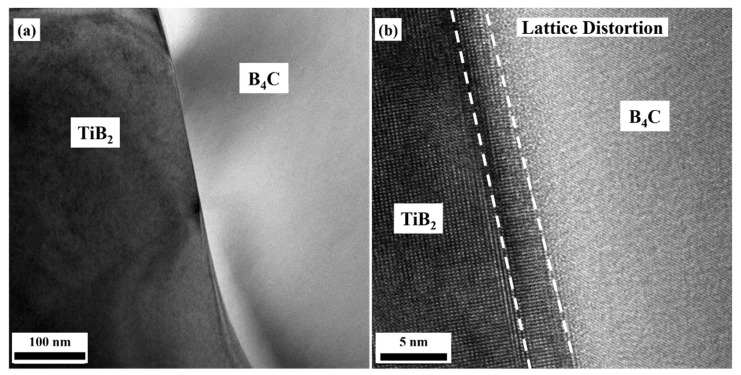
Interface structure between B_4_C and TiB_2_. (**a**) Bright field TEM image, (**b**) HRTEM image.

**Figure 10:**
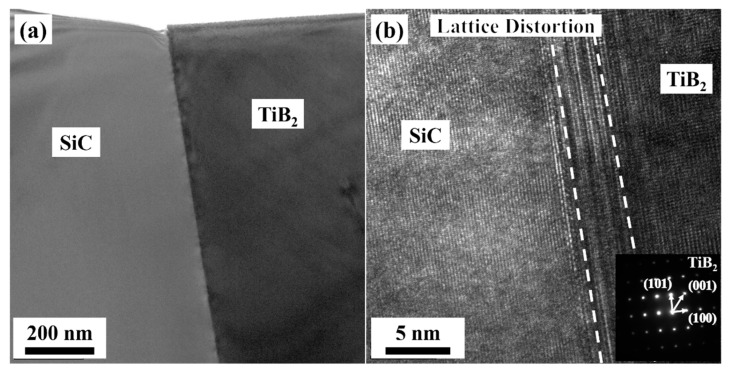
Interface structure between SiC and TiB_2_. (**a**) Bright field TEM image, (**b**) HRTEM image.

Figure 9 and Figure 10 are the bright field and high-resolution TEM images of the interfaces between TiB_2_ and B_4_C, SiC, respectively. As can be seen in the bright field images of Figure 9a and Figure 10a, the interfaces were clean and straight, and there were no other new phases. There were lattice distortion regions with a wide range of 2~3 nm at the interfaces, which were also the transition regions of the lattice structures between TiB_2_ and the other two phases, shown in Figure 9b and Figure 10b. The main reason for the formation of these transition zones may be attributed to the unit cell structure parameters of TiB_2_: a = b = 0.30 nm, c = 0.32 nm, α = β = 90°, γ = 120°. Additionally, the difference of the unit cell structure between B_4_C, SiC, and TiB_2_ was huge. During the sintering process, the transition zones were created to coordinate the arrangement of atoms at the interfaces.

The mechanical and physical properties of the prepared ceramic composites with different TiB_2_ particle sizes are presented in Table 2. With the size of TiB_2_ raw powder decreasing, the relative density and mechanical properties of the prepared ceramic composites all showed a significantly increasing trend. The relative density of the prepared specimens increased, which helped to achieve the excellent mechanical properties of the prepared specimens. The relative density of the BS20N10 sample reached 98.6%, which is the first major requirement to obtain competitive B_4_C/TiB_2_ ceramic composites. The optimized flexural strength, Vickers hardness, and fracture toughness of the BS20N10 sample reached 364 MPa, 30.2 GPa, and 5.47 MPa·m^1/2^, respectively.

The relative density of the BS20N10 sample was high (98.6%) and the grain sizes were fine (about 2.63 μm), which were mainly due to the following four aspects: (1) the 50 nm-sized TiB_2_ particles filled the pores of the green body and increased the density of the green body, being conducive to higher densification upon sintering; (2) with the size of the TiB_2_ powder decreasing, the specific surface energy of the green body was higher than in analogous compositions with coarser grain sizes, which provides a strong driving force for sintering; (3) with the size of TiB_2_ powder decreasing, the amount of the grain boundary increased, resulting in enhanced grain boundary diffusion during the sintering process; (4) TiB_2_ grains on the grain boundaries hindered the movement of the grain boundaries and helped to preserve a fine B_4_C grain size.

The improvement of the relative density and reduction of the grain size of the samples both contributed to obtain high flexural strength. In addition, the shapes of the pores in the BS20N10 were regular polygons or near circles, as shown in Figure 4. According to the fracture mechanics of ceramic materials [31,32,33], these types of the pores could significantly increase the critical value of fracture failure caused by the stress concentration in the sample, and the sample could achieve a high flexural strength.

**Figure 11:**
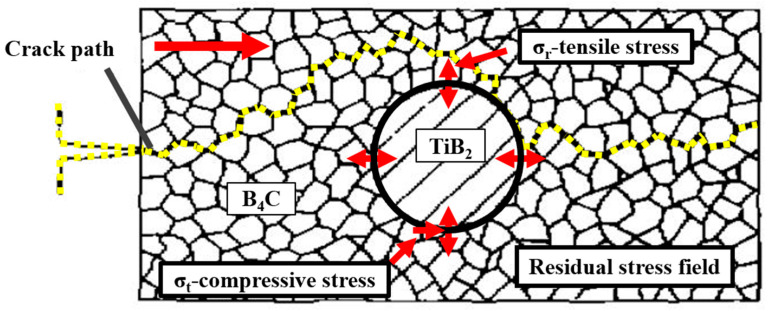
Schematic diagrams of the toughing mechanism by residual stress.

The thermal expansion coefficients of TiB_2_ (8.1 × 10^−6^/°C), B_4_C (4.5 × 10^−6^/°C), and SiC (4.7 × 10^−6^/°C) are quite different [34,35]. During the cooling process, the residual tensile stress fields rise at the interfaces between TiB_2_ and another phase (such as B_4_C or SiC). When the crack enters the residual stress field zone, the crack propagated proceeds in the direction perpendicular to the tensile stress as shown in Figure 11, so that the crack propagation directions can be deflected. The crack deflections and the crack propagation paths are extended, which increase the energy consumption and increase the fracture toughness of the prepared ceramic composite.

## 4. Conclusions

B_4_C/TiB_2_ ceramic composites containing different proportions of submicron and nano TiB_2_ powders were prepared by pressureless sintering at 2100 °C. With the decrease of the particle size of TiB_2_ raw powders, the surface energy of the powder increased significantly and the density of the sintered body increased. During the sintering process, nano-TiB_2_ inhibited the grain growth, increased the number of the grain boundaries, and promoted the densification of the material to 98%. With the size of TiB_2_ powders decreasing, the average grain sizes of the B_4_C/TiB_2_ ceramic composites decreased, and the interfaces between the different phases were strongly bonded, which helped to obtain good mechanical properties. As a result, the B_4_C/TiB_2_ ceramic composite with 20 wt.% submicron and 10 wt.% nano-TiB_2_ addition had a significant improved in mechanical and physical properties. The optimized relative density, grain size, Vickers hardness, flexural strength, and fracture toughness of the sample were 98.6%, 2.63 μm, 30.2 GPa, 364 MPa, and 5.47 MPa·m^1/2^, respectively. Finally, it was illustrated that the sub-fine TiB_2_ powder could control the grain growth in the preparation of the B_4_C/TiB_2_ ceramic composite under the pressureless sintering condition, and was confirmed to be an effective approach to enhance the mechanical properties of B_4_C ceramics.

## Figures and Tables

**Table 1:** Starting composition of BM30, BM10S20, and BS20N10 ceramic composites.

Grade	B_4_C (wt.%)	C (wt.%)	Si (wt.%)	Micron TiB_2_ (wt.%)	Submicron TiB_2_ (wt.%)	Nano TiB_2_ (wt.%)
BM30	60	7	3	30	/	/
BM10S20	60	7	3	10	20	/
BS20N10	60	7	3	/	20	10

**Table 2:** Mechanical properties of the B_4_C/TiB_2_ ceramic composites.

Sample	Relative Density (%)	Flexural Strength (MPa)	Fracture Toughness (MPa·m^1/2^)	Vickers Hardness (GPa)
BM30	90.1 ± 0.2	217 ± 13	3.70 ± 0.19	8.3 ± 0.6
BM10S20	92.6 ± 0.1	288 ± 12	4.46 ± 0.12	12.5 ± 1.1
BS20N10	98.6 ± 0.1	364 ± 9	5.47 ± 0.12	30.2 ± 2.6

## Data Availability

Not applicable.
